# Comprehensive Agreement Analysis of Colorimetric and Turbidimetric Total Protein Assays in Cerebrospinal Fluid

**DOI:** 10.3390/diagnostics16010112

**Published:** 2025-12-29

**Authors:** Raffaella Candeloro, Ilaria Ghidini Begliardi, Alice Lodi, Giovanna Negri, Sara Ghisellini, Massimiliano Castellazzi

**Affiliations:** 1 Department of Neurosciences and Rehabilitation, University of Ferrara, 44121 Ferrara, Italy; raffaella.candeloro@unife.it (R.C.); ila.begliardighidini@edu.unife.it (I.G.B.); alice02.lodi@edu.unife.it (A.L.); 2 Clinical Pathology Unit, “S. Anna” University Hospital, 44124 Ferrara, Italy; g.negri@ospfe.it (G.N.); sara.ghisellini@ospfe.it (S.G.)

**Keywords:** cerebrospinal fluid, total protein, Bland–Altman, concordance correlation coefficient, Cohen’s kappa

## Abstract

**Background/Objectives**: Accurate measurement of total protein (TP) in cerebrospinal fluid (CSF) is crucial for diagnosing various neurological conditions. This study aims to evaluate the concordance between a routine colorimetric method and a recently introduced turbidimetric method for measuring CSF TP. **Methods**: We measured 161 CSF samples using both methods, analyzing the whole population and two subgroups: normal (≤500 mg/L) and pathological (>500 mg/L). Agreement was assessed using Lin’s Concordance Correlation Coefficient (CCC), Bland–Altman, and Deming regression, while clinical concordance was determined with Cohen’s Kappa. **Results**: The concentrations obtained from the two methods did not differ significantly and were well-correlated across the population and subgroups. The CCC for the entire dataset was 0.9881 (substantial agreement), while the Bland–Altman analysis showed a mean bias of 4.467 mg/L. For the “normal” subgroup (n = 97), the CCC was 0.8722 (poor agreement), with a mean bias of 7.668 mg/L. In the “pathological” subgroup (n = 64), the CCC was 0.9858 (substantial agreement) with a mean bias of −3.838 mg/L. Demin regression did not show statistically significant proportional or constant bias in the whole population. However, a stratified analysis revealed a significant negative constant bias in the “normal” subgroup in absence of significant bias in the “pathological” subgroup. Cohen’s kappa was 0.804, indicating substantial agreement. **Conclusions**: Both methods showed substantial agreement for quantifying CSF TP and clinical classification, supporting their potential interchangeability for diagnostic purposes. Nonetheless, laboratories should note the presence of bias, particularly for samples near the clinical cut-off value.

## 1. Introduction

Cerebrospinal fluid (CSF), a transparent, acellular biological fluid, envelops and cushions the brain and spinal cord. Its biochemical profile closely mirrors that of a plasma ultrafiltrate, distinguished by a markedly low protein concentration and a negligible cellular component [1,2].

Access to this crucial fluid is typically facilitated through a diagnostic lumbar puncture (or spinal tap)—a minimally invasive clinical procedure employed for the retrieval of CSF from the spinal subarachnoid space [3]. With the sole exception of direct brain biopsy, CSF analysis constitutes the principal investigative modality available for the diagnosis of (i) inflammatory, (ii) infectious, and (iii) degenerative neurological pathologies, as well as for the identification of (iv) computed tomography negative subarachnoid hemorrhage and (v) leptomeningeal metastases [4,5]. To provide expeditious clinical data, physico-chemical evaluations of the CSF are customarily completed within two hours of specimen collection. These analyses furnish essential parameters concerning the fluid’s clarity, the presence of any chromic discoloration, glucose homeostasis indicators, the enumeration of circulating cells, and the total protein (TP) concentration [6].

CSF-TP concentration is one of the key biochemical parameters routinely assessed in neurological diagnostics. Rather than being a direct indicator of blood–brain barrier integrity, CSF-TP largely reflects the entry of plasma proteins into the CSF via pinocytotic transfer by endothelial cells [7]. It is also considered an important screening test for pathological conditions such as autoimmune and infectious polyneuropathies, viral and bacterial infections, subarachnoid hemorrhage and brain metastases [8]. Accurate and reliable measurement of CSF-TP is therefore essential for clinical decision-making and for establishing appropriate reference intervals [7].

The variety of analytical methods available for measuring CSF total protein necessitates a thorough evaluation of their agreement and interchangeability [9]. While the performance characteristics of individual method are important, it is also crucial to understand the level of agreement between methods when applied to the same samples [8,10]. Notably, McCudden et al. compared results of turbidimetric and colorimetric methods highlighting methodological differences and their impact on clinical interpretation of CSF total protein results [8].

The aim of this study was to evaluate the agreement and interchangeability between two distinct analytical methods for CSF TP quantification.

## 2. Materials and Methods

### 2.1. Study Design

The study was approved by the local ethics committee (protocol 770/2018/Oss/AOUFe) [11]. All procedures followed international guidelines and good clinical practice [12]. Written informed consent was obtained.

Exclusion criteria included CSF white blood cells > 10/μL, presence of discoloration (e.g., presence of haemolysis and xanthochromia) and CSF repeated samples.

The authors, who are non-native English speakers, used Google Gemini (a large language model) exclusively for proofreading, checking grammar, refining syntax, and improving the overall fluency of the English language throughout the final draft of the manuscript. The use of this tool was limited to correcting pre-existing text authored by the researchers; no content or scientific conclusions were generated by the AI. Additionally, the authors used the free reference management tool EndNote Basic to manage, cite, and format the bibliography and references listed in the manuscript.

### 2.2. Sample Collection and Analysis

A total of 161 CSF samples were collected and processed in the same conditions [12]. After centrifugation (2000× *g*, 10 min, 20 °C), supernatants were aliquoted and stored at −80 °C when not immediately analyzed. All samples were handled under standardized conditions [13].

Method A: At the time of lumbar puncture, CSF TP levels were measured as a part of the diagnostic work-up using Beckman Coulter Urinary/CSF Total Protein reagent with Beckman CoulterAU640/AU640e (Beckman Coulter, Brea, CA, USA). This colorimetric test is based on the pyrogallol red-molybdate complex, which binds to the basic amino groups of proteins to form a blue-violet complex, measured photometrically at 600 nm. The test is linear in the range 0.01–2.00 g/L, with a detection limit of 0.005 g/L, and has been validated for CSF samples. Quality control was performed according to the manufacturer’s instructions, and calibration was traceable to a primary human serum albumin standard.

Method B: In March 2025, stored CSF samples were remeasured using a turbidimetric method on the Optilite^®^ analyser (The Binding Site, a Thermo Fisher Scientific company, Birmingham, UK). The Optilite^®^ Total Protein Low Level test (NK061.L.OPT) is a relatively new product designed for high-sensitivity quantification of protein concentrations in CSF and urine. Calibration and control were performed using reagents supplied by the manufacturer.

Method A was used as the reference standard, employing the “normal” threshold of 500 mg/L that was utilized in routine diagnostic at the time of lumbar puncture. Samples were stratified into “normal range” (≤500 mg/L) or “pathological range” (>500 mg/L) accordingly.

### 2.3. Statistical Analysis

Statistical analyses were performed using Prism 10 (GraphPad Software, La Jolla, CA, USA). Distributions of data were checked with Kolmogorov–Smirnov test. Comparisons between non-parametric data were performed using the Wilcoxon test and the Spearman test for correlation. Data with a parametric distribution were analyzed using a *t*-test and a Pearson test for linear correlations.

Agreement between methods was assessed using Lin’s Concordance Correlation Coefficient (CCC) [14], Bland–Altman analysis [15], Deming regression [16], Cohen’s Kappa [17]. The CCC was interpreted according to the range used for medical and biomedical interpretation [18]: >0.99, almost perfect; 0.95–0.99 substantial agreement; 0.90–0.95 moderate agreement; <0.90, poor agreement. Cohen’s Kappa was interpreting following the guidelines for medical diagnostics [19]: 1.00, perfect agreement; 0.81–1.00, almost perfect agreement; 0.61–0.80, substantial agreement; 0.41–0,60, moderate agreement; 0.21–0.40, fair agreement; 0.01–0.20, slight agreement; ≤0, no agreement.

## 3. Results

### 3.1. Pre-Analytical Validation and Analytical Performance Assessment

To ensure the integrity of the results and minimize potential sources of pre-analytical variability, several quality control measures were implemented before the study began. One of the primary concerns addressed was the potential impact of long-term storage and sample handling on analyte stability. All CSF samples analyzed with Method 2 were stored at −80 °C immediately after collection. It is crucial to note that these samples were rigorously kept frozen and did not undergo freeze–thaw cycles before final analysis. This precaution was taken to prevent protein degradation or structural alterations that could compromise measurement accuracy, as repeated temperature fluctuations are known to introduce significant analytical bias.

To further validate the reliability of Method 2 under our specific laboratory conditions, an internal precision study was conducted using three CSF samples. These samples were analyzed multiple times, both within a single analytical run and over several consecutive runs. The results demonstrated excellent analytical stability: the intra-assay coefficient of variation (CV) remained consistently below 5%, while the inter-assay CV remained below 8%. Furthermore, when comparing these results to initial measurements obtained with Method 1, the total deviation remained within 8%. These performance parameters are not only highly satisfactory, but also closely aligned with the technical specifications and performance claims provided by the manufacturers of both diagnostic platforms.

Achieving these levels of precision allowed us to conclude, a priori, that freezing at −80 °C did not adversely affect the quality or immunoreactivity of the analyzed samples. This evidence supports the interchangeability of results regardless of the storage period. To continuously monitor the system’s accuracy, each analytical run for Method 2 included multiple levels of internal quality control (IQC) materials provided by the manufacturer. These controls were strategically placed throughout the analyses to detect any drift or systematic errors.

### 3.2. Cerebrospinal Fluid Total Protein Concentrations

A total of 161 consecutive CSF samples were analyzed blindly from the patient’s diagnosis and demographic data such as gender and age.

Following the assessment of data distribution using the Kolmogorov–Smirnov test, the values obtained by the two investigation methods did not show a normal distribution in the overall population and in the “pathological” concentration subgroup. Conversely, a normal distribution was observed for the data within the subgroup characterized by “normal” CSF TP concentrations (Table 1).

The central tendency of the CSF TP concentrations obtained with the two methods was statistically compared across all defined groups (Table 1). The median CSF TP concentrations did not differ significantly in the entire study population (450 vs. 442.5 mg/L) nor in the two separate subgroups. Specifically, the “normal range” subgroup exhibited highly similar mean concentrations (363.3 vs. 355.6 mg/L), as did the “pathological range” subgroup (median 655 vs. 661 mg/L). These findings collectively demonstrate that the two methods yield statistically comparable results for CSF TP measurement, regardless of the concentration range.

**Figure 1:**
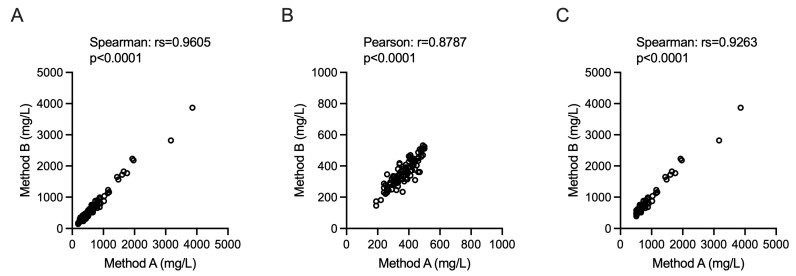
Correlation between values measured using Method A and Method B. Method A was used as the reference standard, employing the “normal” threshold of 500 mg/L that was utilized in routine diagnostic at the time of lumbar puncture. Correlation plots are shown for the entire sample set (Panel (**A**)), the subgroup with “normal” concentrations (Panel (**B**)), and the subgroup with “pathological” concentrations (Panel (**C**)). A strong, statistically significant correlation was observed in all cases (All *p*-values < 0.0001). The type of analysis (Spearman or Pearson) and the coefficient (r or rs) are indicated for each panel.

The measurements obtained from the two analytical methods, Method A and Method B, demonstrated a strong and significant correlation across all examined datasets. The comparison between the two methods using the entire sample cohort (Figure 1A) revealed an extremely robust non-parametric correlation (Spearman’s rs = 0.9605, *p* < 0.0001). This concordance was maintained even when the samples were analyzed within specific concentration subgroups. Specifically, the subgroup of samples with concentrations falling within the “normal” range (Figure 1B) exhibited a significant Pearson correlation coefficient (r = 0.8787, *p* < 0.0001). Similarly, the subgroup with elevated (“pathological”) concentrations (Figure 1C) also showed a very high non-parametric correlation (Spearman’s rs = 0.9263, *p* < 0.0001).

### 3.3. Lin’s Concordance Correlation Coefficient and Bland–Altman Analysis

**Figure 2:**
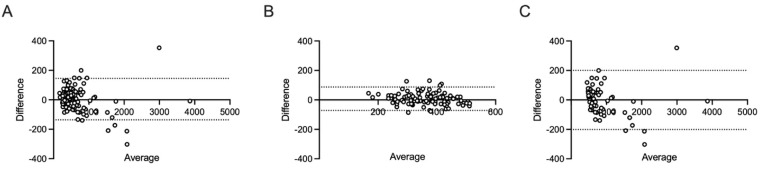
Bland–Altman plot for the comparisons between Method A and Method B for cerebrospinal fluid (CSF) total protein (TP) concentrations (mg/L). The x-axis represents the average of the two methods’ measurements. The y-axis represents the difference between the two methods’ measurements (Method A − Method B). The solid line indicates the mean difference (bias), and the dotted lines represent the 95% Limits of Agreement. Method A was used as the reference standard, employing the “normal” threshold of 500 mg/L that was utilized in routine diagnostic at the time of lumbar puncture. (**A**): overall population (n = 161). (**B**): “normal” samples (CSF TP ≤ 500 mg/L) (n = 97). (**C**): “pathological samples” (CSF TP > 500 mg/L) (n = 64).

The overall CCC calculated for the entire dataset was 0.9881. This high value is highly indicative of a substantial agreement and strong interchangeability between the two methodologies across the full spectrum of measured concentrations. The accompanying Bland–Altman analysis (Figure 2A) further characterized this agreement. It revealed a minimal mean bias of 4.467 mg/L, suggesting that the methods, on average, produce very similar results. The dispersion of the differences was reflected by a standard deviation (SD) of bias of 71.79. The 95% Limits of Agreement (LoA) ranged from −136.0 to 145.0 mg/L. This range defines the interval within which 95% of the differences between the two methods are expected to fall.

A contrasting pattern emerged when the analysis was restricted to the subgroup of samples with CSF total protein (CSF TP) concentrations ≤ 500 mg/L (the “normal” range; n = 97). Within this lower concentration range, the CCC was 0.8722. According to established criteria for agreement, this value indicates a poor level of agreement. The Bland–Altman plot for this subgroup (Figure 2B) demonstrated a mean bias of 7.668 mg/L (SD of bias = 40.67). Crucially, the 95% LoA were significantly tighter, spanning from −72.04 to 87.38 mg/L. While the LoA range is smaller, the lower CCC value suggests that at these specific concentrations, the agreement is less robust than across the entire range.

Conversely, for the subgroup containing samples with CSF TP concentrations > 500 mg/L (n = 64), the methods once again exhibited a strong performance. The CCC was calculated to be 0.9858, which strongly reaffirms a substantial agreement in the clinically relevant high-concentration range. The Bland–Altman analysis (Figure 2C) showed a slight negative mean bias of −3.838 mg/L, indicating that Method B tended to report marginally lower values than Method A in this group. The higher variability associated with these elevated concentrations resulted in a standard deviation of bias of 102.4 and consequently, the widest 95% LoA, which extended from −201.2 to 200.4 mg/L.

### 3.4. Deming Regression Analysis

Deming regression was performed to assess the agreement between Method A and Method B for measuring CSF total protein (Table 2).

The overall analysis, conducted across all 161 samples, yielded an equation that demonstrated not statistically significant proportional or constant bias. This was confirmed because the 95% confidence intervals (CI) for both the slope and the Y-intercept included the ideal values of 1.000 and 0.000, respectively. However, a stratified analysis, summarized in Table 2, revealed significant concentration-dependent differences. In the normal range (≤500 mg/L, N = 97), a significant proportional bias and a significant negative constant bias were observed, as neither the 95% CI for the slope nor that for the Y-intercept included the ideal values. Conversely, in the pathological range (>500 mg/L, N = 64), the 95% CI for the slope and intercept both included the ideal values, demonstrating a statistically non-significant bias in the detection of elevated protein levels.

### 3.5. Cohen’s Kappa Concordance Analysis

To assess the concordance of the two methods in classifying samples into clinical categories, all 161 samples were dichotomized as “normal” (≤500 mg/L) or “pathological” (>500 mg/L). Cohen’s Kappa was calculated to be 0.804 (95% CI = 0.710 to 0.898). This value, which falls into the substantial agreement range, indicates a good concordance between the two methods in assigning samples to the normal or pathological category, well beyond what would be expected by chance. The observed disagreements (6 samples where Method A classified as normal and Method B as pathological; 9 samples where Method A classified as pathological and Method B as normal) represent a small percentage of total samples, primarily occurring near the classification threshold.

## 4. Discussion

Our analysis revealed a strong overall concordance between the two methods across the full range of protein concentrations. This agreement was supported by non-significant differences in mean values and a statistically non-significant overall bias in the Deming regression, confirming that both methods are broadly consistent. However, a crucial finding emerged when data were stratified: while the agreement remained excellent for pathological samples (>500 mg/L), it significantly deteriorated for samples within the normal range (≤500 mg/L). Specifically, the statistical analysis indicated the presence of both a proportional and a constant bias in the low concentration subgroup. This result is consistent with inter-method bias previously reported in the literature, such as that described by McCudden et al., who noted a comparable difference of 40 mg/L between turbidimetric and colorimetric tests [8]. Such discrepancies, particularly at low protein concentrations, indicate that some degree of method-dependent variation may persist, especially near the lower analytical range. From a clinical perspective, the utility of both methods remains high, as shown by the Cohen’s Kappa analysis, which demonstrated substantial agreement in classifying samples as “normal” or “pathological.” This strong clinical agreement suggests that the statistical biases primarily affect values far from the clinical cut-off, limiting the potential for significant misclassification.

An alternative approach to manage this uncertainty in clinical practice could be to define a “grey zone” around the clinical decision limits, representing the analytical uncertainty within which results may vary by approximately ± the observed bias. Specifically, the bias identified in the normal range in our study (41.28 mg/L) is consistent with previously reported values (approx. 40 mg/L) [8]. These findings suggest that establishing a grey zone of ±50 mg/L, conservatively rounded up, around the 500 mg/L positivity threshold could provide a more robust framework for clinical interpretation.

The interpretation of our results is primarily limited by the lack of a recognized gold standard, which remains a key limitation of this study. This lack of a definitive reference method, and the resulting paucity of reference literature, makes it difficult to cross-compare our data with established reference values. This framework is particularly relevant when addressing the observed bias at lower concentrations. Although both assays are validated for routine clinical use, our study was unable to definitively investigate the potential role of matrix effects as a source of this discrepancy. Notably, such interferences had previously been hypothesized but not explored by other authors [8]; similarly, our experimental design did not allow for a more thorough characterization of these effects. Until a universal reference method is established, the precise contribution of sample matrix to analytical variability will remain a challenge, requiring cautious interpretation of results near clinical decision thresholds.

From a practical perspective, given the consistent overall agreement observed between the two techniques, the choice between them can ultimately be guided by the laboratory’s specific diagnostic needs and operational setting. Method A is a rapid, ready-to-use colorimetric assay and is already widely utilized in routine practice. In contrast, Method B, whose reagents have recently been introduced on the market, offers a significant advantage by allowing for the simultaneous evaluation of additional CSF biomarkers. These markers may include albumin, immunoglobulins, and free light chains. Specifically, the assessment of albumin concentrations in both serum and cerebrospinal fluid is crucial, as it enables the calculation of the QAlb (CSF-to-serum albumin ratio), which provides essential information regarding blood-cerebrospinal fluid barrier permeability [20]. Furthermore, IgG quantification is routinely performed to offer a quantitative measure of intrathecal antibody synthesis [21]. Lastly, the measurement of kappa free light chains (k-FLC) and the subsequent calculation of the k-FLC Index have recently been proposed as a valuable supportive test in the diagnosis of multiple sclerosis [22,23].

## 5. Conclusions

Both methods demonstrated substantial agreement in quantifying TP in CSF across all ranges tested and in clinical classification. These results support their interchangeability for diagnostic purposes in standard clinical laboratory procedures. However, laboratories should be aware of the existence of bias between the various methods, especially when interpreting results for samples with TP concentrations close to the cutoff value.

## Figures and Tables

**Table 1:** Descriptive statistics of cerebrospinal fluid total protein concentrations evaluated using two methods: A (Colorimetric) and B (Turbidimetric). Method A was used as the reference standard, employing the “normal” threshold of 500 mg/L that was utilized in routine diagnostic at the time of lumbar puncture. Accordingly, for both methods, data are presented for the whole population (total) and stratified into two groups: ≤500 mg/L, normal samples, and >500 mg/L, pathological samples. The Kolmogorov–Smirnov test was used to assess the normality of distribution for each variable.

	Method A Total	Method B Total	A Vs B *p*	Method A “Normal Range”	Method B “Normal Range”	A Vs B *p*	Method A “Pathological Range”	Method B “Pathological Range”	A Vs B *p*
	(mg/L)	(mg/L)		(mg/L)	(mg/L)		(mg/L)	(mg/L)	
Number of values	161	161		97	97		64	64	
Minimum	190.0	145.2		190.0	145.2		510.0	391.5	
25% Percentile	340.0	333.8		300.0	291.7		560.0	538.9	
Median	450.0	442.5	0.1965 ^a^	360.0	355.1		655.0	661.0	0.8865 ^a^
75% Percentile	600.0	591.6		425.0	421.8		880.0	894.8	
Maximum	3860	3868		500.0	532.0		3860	3868	
Mean	569.7	565.2		363.3	355.6	0.0665 ^b^	882.5	882.9	
Std. Deviation	459.5	470.8		78.46	84.80		601.6	617.9	
Std. Error of Mean	36.22	37.10		7.966	8.610		75.20	77.23	
Passed normality test (*p*) ^c^	No (<0.0001)	No (<0.0001)		Yes (>0.1000)	Yes (>0.1000)		No (<0.0001)	No (<0.0001)	

^a^ Wilcoxon matched-pairs signed rank test; ^b^ Paired *t* test; ^c^ Kolmogorov–Smirnov test.

**Table 2:** Deming Regression Analysis for Method Comparison in all samples and in the normal and pathological range subgroups. Method A was utilized as the reference standard for this comparison, employing the established “normal” threshold of 500 mg/L that was in use for routine diagnostic purposes at the time the lumbar punctures were performed.

Sample Group	N	Regression Equation (Y = mX + b)	Slope (m) (95% CI)	Y-Intercept (b) (mg/L) (95% CI)	Agreement Conclusion
All Samples	161	Y = 1.025X − 18.57	1.025 (0.9416 to 1.108)	−18.57 (−58.71 to 21.58)	No significant bias
Normal Range (≤500 mg/L)	97	Y = 1.093X − 41.28	1.093 (**1.002 to 1.183**)	−41.28 (**−75.02 to −7.536**)	Significant Proportional & Constant Bias
Pathological Range (>500 mg/L)	64	Y = 1.027X − 23.80	1.027 (0.9155 to 1.139)	−23.80 (−105.4 to 57.81)	No significant bias

Bold 95% confidence intervals (CIs) indicate that the ideal value (1.000 for Slope, 0.000 for Y-Intercept) is excluded.

## Data Availability

The datasets used and analyzed during the current study are available from the corresponding author upon reasonable request.

## Abbreviations

The following abbreviations are used in this manuscript:

CCC	Lin’s Concordance Correlation Coefficient
CI	Confidence intervals
CSF	Cerebrospinal fluid
LoA	Limit of Agreement
SD	Standard deviation
TP	Total protein
